# The National Insurance Bill

**Published:** 1911-06-10

**Authors:** 


### THE NATIONAL INSURANCE BILL.

BEFORE the House of Commons adjourned for the Whitsuntide recess Mr. Lloyd George answered a number of questions asked with the object of clearing up doubtful points in the National Insurance Bill. Many of these dealt with the position of medical practitioners.

Replying to a question asked by Mr. Astor, the Chancellor of the Exchequer said the average fee paid to German practitioners by the sickness funds of all kinds in 1909 was 5s. 8d. per person insured. This fee was exclusive of drugs, but included attendance upon uninsured members of families where the rules of a fund provided for such attendance. The average fee paid to doctors by the parochial funds, which as a rule do not provide family attendance, was 4s. per person insured. It was really very difficult to ascertain how much was paid for drugs, because in Germany the amount very often included the cost of expensive appliances. The purpose of another question was to inquire whether consideration could be taken in the scheme of the position of a practitioner in a scattered district who, for a fee of 6s., might often have to visit a case so distant as to occupy five or six hours of time. The Chancellor replied that this would be a matter for the practitioners concerned to arrange with the society or health committee, subject to the arrangement being satisfactory to the Insurance Commissioners.

Mr. Hodge asked the Chancellor (1) whether the 6s. per head which he proposed to set aside for medical assistance and drugs in respect to each insured person would apply to those insured voluntarily through the Post Office as well as to employed contributors; (2) whether he had considered the desirability of allowing insured persons under his insurance scheme to retain their own doctors, subject to the provisions of Clause 14. In reply Mr. Lloyd George said that in the actuarial calculations the same sum was allocated to medical attendance for the Post Office contributions as for the others, but power is given by Clause 14 to the local health committee to supplement this, with the consent of the Treasury and the county council or county borough council, from the Exchequer and from local rates. In answer to a further question, he said the Insurance Commissioners would have power under the Bill to ensure fair conditions of payment to medical practitioners for their services to insured persons. If the 6s. turned out to be insufficient, the surplus of 2s. 6d. per member provided in the finances of the scheme could be drawn upon. With regard to persons insured under the Post Office, it was obvious that 6s. would not cover the medical attendance in those cases, inasmuch as it was an insurance on a bad life. Several questions related to the maternity benefit, and the effect of the Chancellor's replies was that the maternity benefit of 30s. was intended to cover cost of doctor and midwife where necessary, and that, while there is nothing in the Bill which necessarily involves any restriction upon a woman's choice of medical attendant or midwife, the matter was one for the approved society or the local health committee, as the case may be, to arrange. Replying to a question asked by Mr. Pike Pease, the Chancellor explained what steps he had taken to seek advice of those who represented general medical practitioners; he had seen representatives of the British Medical Association twice—once before and once since the introduction of the Bill. He had also seen representatives of the General Medical Council, and had consulted from time to time other representatives of the medical profession.

Notices of a number of amendments to be moved in Committee have already been put on the paper, but few of these affect the position of the medical profession under the proposed scheme; one of them provides that no woman who is herself entitled to receive maternity benefit shall thereby be disqualified from receiving in addition such sickness, disablement, sanatorium, medical, or other benefit to which she is otherwise entitled; another proposes to provide "surgical instruments, spectacles, and other necessary appliances" as well as medicine, for insured persons. When the House reassembles, in accordance with a suggestion of the Chancellor of the Exchequer committees representative of the different parties are to meet separately and to be taken into consultation by the Government; the object is to establish a kind of informal clearing-house of information and interchange of ideas, which, it is hoped, will greatly simplify the discussion of details of the Bill in Committee.

